# On the Frequency of Consumption in the Island of Malta

**Published:** 1817-02

**Authors:** Thomas Sutton


*For the London Medical and Physical Journal.*

### *On the frequency of Consumption in the Island of Malta;* by Dr. Thomas Sutton.

I HAVE been favoured by Sir James M'Grigor with two returns of the relative mortality among the troops in Malta and Jamaica; and hope soon, through the same channel, to be favoured with more such documents from other parts of the world. It may be recollected by most of your readers, as have interested themselves on the subject of the discussions on Consumption which have appeared lately in the Medical and Physical Journal, that Malta has been mentioned as a climate in which consumption appears rarely. I, however, did not assent to this from the circumstance of the disease having been observed to be common in Sicily. The return alluded to is positive evidence on the subject, and shows the mortality by consumption in Malta to be great. The total deaths among the troops there, including one year to last November, was 51, of which 10 died by pulmonary consumption.

From the return of mortality in Jamaica, it appears, that, in the year 1813, the total number of deaths among the troops was 370, of which 33 died by consumption. In 1814, the total number of deaths was 314, of which 38 died by consumption, making the ratio of deaths by consumption to the general amount as less than one in ten, which, taken in the relative way, cannot warrant the conclusion that it is an unfrequent disease, as has been stated in the Medical and Physical Journal. But, on taking another view of the subject, we shall find this last account of deaths by consumption to exceed the relative number of deaths in Malta by the same disease. The number of troops in Jamaica, from which this account of mortality is taken, never exceeded 4800 in the year 1813. The troops in Malta, during a similar period also, never were one-fifth less than those in Jamaica. Stating, therefore, the mortality by consumption in Malta to be, during this period, twelve, under the supposition—had the troops in Malta been equal to those in Jamaica, that such would have been the ratio of consumption in the former place. Then the ratio of mortality by consumption, for the same number of troops, would be as 12 in Malta to 33 in Jamaica; that is, almost one-third more, bringing the ratio of consumption in Jamaica very high indeed. The ratio of mortality in Jamaica to that in Malta, among the troops, is as 6 to 1, or nearly.

*Croom's Hill, Greenwich; Jan. 17, 1816.*

---

We must apologise to Dr. Sutton for our inattention to the above, which which we received according to the date. It was, by some accident, mislaid, and only discovered by accident amongst some other papers.—EDIT.

---

*For the London Medical and Physical Journal.*

